# Brain and Behavior in Persuasion: The Role of Affective‐Cognitive Matching

**DOI:** 10.1111/psyp.70088

**Published:** 2025-06-08

**Authors:** S. Di Plinio, A. Aquino, G. Haddock, F. R. Alparone, S. J. H. Ebisch

**Affiliations:** ^1^ Department of Neuroscience, Imaging and Clinical Sciences Chieti‐Pescara University Chieti Italy; ^2^ Institute of Advanced Biomedical Technologies (ITAB), Chieti‐Pescara University Chieti Italy; ^3^ Department of Health Sciences Magna‐Graecia University Catanzaro Italy; ^4^ School of Psychology Cardiff University Cardiff UK

**Keywords:** affective and cognitive orientation, affective‐cognitive matching, elaboration likelihood model, functional connectivity, persuasion

## Abstract

The effectiveness of persuasive messages often depends on how their affective or cognitive content aligns with recipients' predispositions for processing such information. Individual differences in the need for affect (NFA) and need for cognition (NFC) influence engagement with affective or cognitive appeals, but the interplay between intrinsic brain connectivity and these predispositions in shaping persuasive outcomes remains underexplored. This study advances understanding of the affective‐cognitive matching effect by integrating intrinsic (resting‐state) and extrinsic (task‐based) brain‐behavior relationships. Using resting‐state and task‐based functional magnetic resonance imaging (fMRI), we investigate how NFA and NFC align with intrinsic brain network properties and influence behavioral and neural responses to affective and cognitive persuasive messages. We employ intrinsic connectivity metrics, such as participation coefficient (cross‐network communication) and within‐module degree (within‐network communication), to capture resting‐state network dynamics not examined in previous studies. Our results reveal that key regions within the frontoparietal network, which is central to attention, decision‐making, and executive functions, play pivotal roles in processing persuasive messages based on participants' motivational orientations. Specifically, affective‐oriented individuals exhibit greater neural engagement with congruent affective messages, while cognitive‐oriented individuals show intensified engagement under incongruent conditions—a novel finding extending beyond prior research. These findings expand the affective‐cognitive matching effect to include intrinsic neural dimensions, highlighting how resting‐state brain connectivity primes responses and modulates task engagement according to motivational predispositions. This integrative approach supports the Elaboration Likelihood Model by elucidating distinct neural pathways in persuasion and offers actionable insights for tailoring persuasive strategies to individual affective and cognitive orientations.

## Abstract 2

This study reveals how intrinsic brain connectivity aligns with affective‐cognitive predispositions, shaping brain and behavioral responses to persuasive messages. By linking neural patterns with individual motivation, this research offers insights for tailoring persuasive strategies, extending the affective‐cognitive matching effect and advancing existing psychological models in persuasion science.

## 1 Introduction

Researchers have long recognized that the content of attitudes can be organized around affective and cognitive components (Cacioppo et al. 1989; Insko and Schopler 1967; Rosenberg and Hovland 1960; Maio et al. 2018; Svenningsson et al. 2021). Typically, the term *affect* has been used to refer to the positive or negative feelings and emotions that an attitude object evokes in the evaluator, whereas the term *cognition* describes beliefs about the positive or negative attributes of an attitude object (e.g., Breckler 1984; Conner et al. 2021; Crites et al. 1994; Ostrom 1969; Haddock et al. 2008). The categorization of persuasive appeals as affective or cognitive in nature extends beyond the mere content of attitudes and influences how they are designed (Haddock and Maio 2019; See et al. 2008).

Numerous studies have tested the hypothesis that persuasive messages are more impactful when their affective or cognitive content aligns with the recipient's predisposition to process affective or cognitive information—a phenomenon known as the affect‐cognition matching effect (Edwards 1990; Fabrigar and Petty 1999; Giammusso et al. 2022). In this regard, individual differences in the motivation to seek out and use affective or cognitive information can be assessed through individual differences in the need for affect (NFA; Maio and Esses 2001) and the need for cognition (NFC; Cacioppo and Petty 1982). NFA reflects the extent to which individuals approach or avoid emotion‐inducing situations, valuing emotions as useful for shaping judgments and behavior. In contrast, NFC reflects a tendency to engage in and enjoy effortful cognitive activities. Both NFA and NFC influence information acquisition and processing (Haddock and Maio 2019). For instance, in the social perception domain, individuals high in NFA evaluate warmth‐related traits more positively, while individuals high in NFC emphasize competence in their evaluations (Aquino et al. 2016). This suggests that NFA and NFC can predict social evaluations toward others (Crites et al. 1994; Fabrigar and Petty 1999; Gharib et al. 2022). In the context of the affective‐cognitive matching effect, Haddock et al. (2008) demonstrated that individual differences in NFA and NFC predicted receptivity to affective or cognitive appeals, with greater attitude change observed when persuasive messages matched recipients' affective or cognitive orientation.

To further understand how these individual differences manifest at the neural level, recent neuroscientific research has begun to explore the brain mechanisms underlying the affective‐cognitive matching effect. Recent advances in neuroscience have underscored the significance of distinguishing between intrinsic and extrinsic brain and behavioral properties in understanding affective and cognitive processes (Biswal et al. 2010; Raichle 2010; Aquino et al. 2020; Di Plinio et al. 2023). Intrinsic brain properties refer to the brain's inherent functional organization, as observed during resting‐state, task‐free fMRI scans. These scans capture the brain's baseline network architecture, revealing patterns of connectivity when a person is not engaged in a specific task (Fox and Raichle 2007; Raichle 2015). Intrinsic connectivity networks show properties like global brain modularity and intermodular connectivity that are predictive of individual differences in behavior, such as sense of agency or susceptibility to psychosis‐like experiences (Di Plinio et al. 2020; Di Plinio and Ebisch 2022). These findings suggest that resting‐state brain dynamics are linked to individual predispositions that can influence how people process task‐related stimuli (Rosazza and Minati 2011; Tsvetanov et al. 2016). Essentially, the information exchange across specific networks can predispose individuals to respond to external demands (Rasero et al. 2018). This supports the idea that intrinsic networks support the brain's responses to specific tasks by organizing neural resources in advance.

Graph‐theoretical metrics, such as the participation coefficient, which measures how extensively a brain region interacts across multiple networks, and the within‐module degree, which assesses connectivity within specific networks, are used to quantify these intrinsic connectivity patterns (Rubinov and Sporns 2010; Power et al. 2010). Intrinsic behavioral properties, including NFA and NFC, are relatively stable individual differences that influence how individuals typically approach and process affective and cognitive information (Cacioppo and Petty 1982; Maio and Esses 2001). For instance, Di Plinio et al. (2023) found that intrinsic connectivity patterns within the frontoparietal module, as measured by participation coefficients, are linked with an individual's affective or cognitive orientation. These findings suggest that intrinsic brain connectivity may underlie the motivational orientations (NFA and NFC) that guide how individuals process persuasive messages, potentially influencing their neural responses and behavioral evaluations during persuasion tasks.

In contrast to intrinsic connectivity, extrinsic brain properties involve neural responses elicited during task performance, captured through task‐based fMRI (Poldrack 2007). Prior work has examined how task‐related brain activity supports persuasive processing (e.g., Falk et al. 2015; Lee et al. 2019). However, these studies have typically focused on predefined regions of interest or clinical samples, and have not explicitly examined how baseline connectivity patterns may predispose individuals to engage with persuasive content. By integrating intrinsic and extrinsic approaches within the same framework, our study extends this literature by investigating how individuals' resting‐state brain connectivity and affective‐cognitive orientations jointly influence neural and behavioral responses to persuasive messages (Tavor et al. 2016). This integration is particularly relevant for studying the affective‐cognitive matching effect, as it offers a neurocognitive basis for understanding how intrinsic predispositions are associated with evaluative responses to persuasive message content (Cacioppo et al. 1996).

Further evidence suggests that matching persuasive messages to an individual's orientation enhances self‐relevance and message processing. For example, greater activation in the ventromedial prefrontal cortex (VMPFC)—a region associated with self‐related processing—has been observed when individuals process health‐related persuasive content, particularly under conditions of self‐affirmation (Falk et al. 2011; Northoff and Hayes 2011). This enhanced self‐relevance can increase motivation to engage with the message, leading to deeper processing (Petty and Cacioppo 1986). Moreover, matching information is typically processed with greater depth than non‐matching information, resulting in more persistent attitude change when arguments are cogent (Petty and Wegener 1998; Wolfe and Kurby 2016).

The existence of matching and nonmatching channels has a parallel in the Elaboration Likelihood Model (ELM), wherein whether a person is influenced more by the substance of the message (central route) or by external cues (peripheral route) depends on their motivation and ability to process the message. According to this framework, when the degree of elaboration is not constrained, self‐relevance plays a pivotal role in increasing recipients' motivation to process message arguments carefully (Petty and Cacioppo 1986; Haugtvedt et al. 1992). Haddock et al. (2008) reported that individual differences in affective or cognitive orientation predicted the amount of information correctly recognized from matching messages, indicating deeper information processing. Further, the persistence of the matching effect over time may differ between affective and cognitive routes. Affective matches might require less processing time to yield persistence due to inherent emotional engagement (Rocklage and Luttrell 2021; Giner‐Sorolla 2004), while cognitive matches may involve more extensive analytical processing, including generating supportive thoughts, leading to persistent attitude change through cognitive elaboration (Briñol and Petty 2018; Teeny et al. 2020). Our study builds upon these findings by examining how intrinsic brain states interact with affective‐cognitive orientations during exposure to affective‐cognitive persuasive content.

### 1.1 The Study Contribution

Despite extensive research on the affective‐cognitive matching effect, the neural mechanisms underlying the effect remain unclear. Prior research has established that intrinsic and task‐based connectivity share substantial network architecture (Smith et al. 2009), while also showing state‐dependent reconfigurations in specific regions (e.g., precuneus) depending on cognitive demands (Utevsky et al. 2014). These findings support the relevance of jointly examining rest and task states, as both stable and context‐sensitive features may contribute to individual variability in neural processing of persuasive messages. Specifically, it is unknown how intrinsic brain dynamics contribute to extrinsic processing of persuasive messages, how this relates to individual orientations, and how matching and nonmatching channels are involved in determining behavioral outcomes. To address this gap, we investigate both intrinsic and extrinsic brain‐behavior relationships using functional magnetic resonance imaging (fMRI). By examining resting‐state fMRI data, we can identify intrinsic brain connectivity patterns associated with individual differences in NFA and NFC, which may predispose individuals to process affective or cognitive information in specific ways (Di Plinio et al. 2023). Task‐based fMRI allows us to measure extrinsic neural responses as participants engage with affective and cognitive persuasive messages. By analyzing changes in neural activation during the task and collecting participants' evaluations of the messages, we can assess how message content and individual orientations interact to influence persuasion.

The integration of these methodologies enables us to investigate whether intrinsic brain‐behavior relationships predict extrinsic brain‐behavior responses. Specifically, we aim to study if individuals with certain intrinsic connectivity patterns and orientations exhibit corresponding neural responses and evaluations during message processing, thus providing a neural basis for the affective‐cognitive matching effect. This is important because identifying such neural signatures would not only help elucidate the mechanisms behind the affective‐cognitive matching effect but also expand our understanding of how baseline brain states predispose individuals to process information in ways that align with their intrinsic orientations. While prior research has examined task‐based and, more recently, resting‐state connectivity in persuasive or social contexts (e.g., Falk et al. 2015; Lee et al. 2019), to our knowledge, no study has yet integrated intrinsic and extrinsic connectivity measures together with motivational orientations and behavioral evaluations in the same framework. Therefore, this study provides an integrative perspective that advances the field by bridging the gap between resting‐state and task‐based neural mechanisms and their implications for persuasion.

Our approach integrates four levels of information processing, namely resting‐state connectivity, task‐evoked activity, individual affective‐cognitive orientation, and explicit message evaluations, to provide an innovative multidimensional analysis. Our methodological approach advances beyond our prior studies by integrating these measures to assess how intrinsic brain states may prime individuals for differential processing of affective and cognitive persuasive messages.

### 1.2 Aims and Hypotheses

Grounded in the ELM (Petty and Cacioppo 1986) and the structural matching effect (Aquino et al. 2020), the present study investigates how individual motivational orientations interact with brain connectivity patterns, both at rest and during task engagement, in relation to responses to affective versus cognitive persuasive messages. We aim to clarify how intrinsic brain states are associated with individual tendencies to engage with different types of persuasive content, and how this alignment correlates with both neural and behavioral responses.

First, based on prior theory (Petty and Wegener 1998; Haddock et al. 2008; Fabrigar and Petty 1999), we hypothesize that individual differences in motivational orientations (NFA vs. NFC) will modulate neural responses to persuasive messages. Specifically, drawing upon existing theoretical perspectives rather than empirical outcomes, we hypothesize that affective‐oriented individuals (higher NFA relative to NFC) will exhibit greater neural engagement in response to affective messages, reflecting congruence between their motivational predispositions and message content. On the other hand, cognitive‐oriented individuals (higher NFC relative to NFA) will engage neural resources differently, potentially reflecting deeper elaboration processes when processing affective messages (incongruent with their motivational orientation).

Second, based on previous evidence indicating that resting‐state connectivity patterns prime neural responses to task demands (Di Plinio et al. 2023; Tavor et al. 2016), we hypothesize that intrinsic brain connectivity, particularly within highly integrative regions (high participation coefficient nodes), will interact with individual motivational orientations to influence task‐related neural responses.

By testing these hypotheses, we aim to explore the neural correlates of the affective‐cognitive matching effect, providing insights into how intrinsic brain states are associated with task‐related neural responses and persuasion‐related evaluations.

## 2 Methods

### 2.1 Participants

Thirty‐five healthy Italian adults (20 women and 15 men; mean age = 25.2 years, SD = 3.4) participated in the study. All participants were right‐handed, had normal or corrected‐to‐normal vision, and had no history of psychiatric or neurological disorders or contraindications for MRI scanning. The study was approved by the Comitato Etico delle Province di Chieti e Pescara e dell'Università degli Studi “G. d'Annunzio” di Chieti‐Pescara (Protocol No. 898) and conducted in accordance with the Declaration of Helsinki (2013). Written informed consent was obtained from all participants prior to participation.

The sample in this study matched standard sizes in individual differences research in cognitive neuroscience and fMRI studies (Yarkoni 2009; Turner et al. 2018). Additionally, it balanced statistical power with the constraints of fMRI data collection and processing, which are resource‐intensive. While power analyses for fMRI are complex due to the high dimensionality of neuroimaging data, studies indicate that samples of this size are appropriate for identifying stable connectivity patterns and task‐related activations (Dubois and Adolphs 2016). To mitigate variability and enhance the reliability of our findings, we used robust statistical techniques, including mixed‐effects models and effect size reporting, in line with current recommendations for fMRI studies (Mumford and Nichols 2008). This sample size thus allows for adequate sensitivity to detect meaningful associations between intrinsic connectivity, task‐evoked activity, and affective‐cognitive orientation, providing a rigorous foundation for our hypotheses while adhering to standard practices in fMRI research.

The study follows our first two experiments, which focused respectively on task‐related fMRI data (Aquino et al. 2020, examined the neural correlates of the structural matching effect on task data) or resting‐state fMRI data (Di Plinio et al. 2023; studied the predictability of choices using machine learning on resting‐state data). The present research includes a larger sample size and incorporates *both* resting‐state and task‐evoked fMRI data. Additionally, our study integrates brain and behavioral measures to investigate how affective‐cognitive orientations relate to neural patterns and individual choices in response to affective and cognitive persuasive messages.

Unlike our prior work focusing on stimulus‐evoked activity, we examined how intrinsic connectivity patterns at rest align with individual affective‐cognitive orientations, thus extending the affective‐cognitive matching effect to resting‐state neural dynamics.

### 2.2 Stimuli

Affective and cognitive persuasive messages were meticulously crafted based on real advertisements, resulting in 20 affective and 20 cognitive messages for various consumer products. Each message consisted of five sentences, with affective messages emphasizing emotions and sensations (e.g., “The soft wool of the pullover ‘Tender’ gives a fresh scent all day”) and cognitive messages highlighting product features and qualities (e.g., “The new full‐resistant pullover is made with 100% merino wool”). All messages were designed to elicit positive reactions to control for valence confounds.

To ensure the effectiveness of these messages, a preliminary validation process was conducted. Initially, 64 participants rated each message on its affective‐cognitive content and credibility. Messages containing self‐references (e.g., “The pullover Tender cuddles you in a warm hug”) were compared to those without self‐references (e.g., “The pullover Tender cuddles who wears it in a warm hug”) to assess the impact of self‐relevance on content perception. The results confirmed that self‐referenced messages significantly enhanced the differentiation between affective and cognitive content. Consequently, the 10 most distinct affective and 10 most distinct cognitive self‐referential messages were selected for the main study. Further validation with an additional 22 participants confirmed that the selected messages effectively differentiated affective and cognitive content without differences in credibility or length.

### 2.3 Pre‐MRI Behavioral Measures

Prior to fMRI scanning, participants' levels of NFA and NFC were assessed. NFA was measured using the short version of the NFA scale (Appel et al. 2012), which consists of 10 items evaluating the motivation to approach and avoid emotions. Participants responded on a 7‐point scale (1 = totally disagree; 7 = totally agree), and the NFA score was calculated by summing responses after reverse‐scoring avoidance items.

NFC was assessed with the 18‐item NFC scale (Cacioppo and Petty 1982), where participants rated statements on a 7‐point scale (1 = extremely uncharacteristic; 7 = extremely characteristic). The NFC score was derived by summing responses after reverse‐scoring negatively keyed items.

To capture personal orientation toward affect and cognition, we computed an Orientation score by subtracting standardized NFC scores from NFA scores (Orientation = Z_NFA_—Z_NFC_). A higher Orientation score indicates a greater reliance on affect, while a lower score reflects a greater reliance on cognition. This difference score approach enhances the interpretability of individual differences in affective‐cognitive orientation and supports robust statistical modeling.

### 2.4 MRI Data Acquisition

Imaging data were collected using a 3 Tesla Philips Achieva X Series MRI scanner at the Institute of Advanced Biomedical Technologies (ITAB) in Chieti, Italy. A sensitivity‐encoding eight‐channel brain coil was utilized to ensure high‐quality imaging, and head motion was minimized with foam padding and surgical tape. Participants interacted with the task using a response pad fixed to the scanner bed, allowing keypresses with their right index and middle fingers. An initial T1‐weighted anatomical image (3‐D TFE pulse sequence) was acquired with the following parameters: field of view = 240 mm; voxel size = 1 mm^3^; TR = 8.1 ms; TE = 3.7 ms. Subsequently, two resting‐state runs (234 volumes for each run) and two task fMRI runs (404 and 397 volumes, respectively) were acquired using a T2* weighted EPI sequence with TR = 1.8 s; TE = 30 ms; number of slices = 35; slice thickness = 3.5 mm; in‐plane voxel size = 3 mm^2^; field of view = 228 × 122 × 240 mm; flip angle = 85°.

### 2.5 MRI Experimental Procedure

Following the assessment of NFA and NFC, all participants underwent an fMRI scanning session, which included both resting state and task based.

With respect to the resting‐state fMRI, participants completed two resting‐state runs, each lasting approximately 6 min. During these runs, they were instructed to focus on a white fixation cross displayed on a black screen with their eyes open. Compliance was monitored via a video camera positioned in the MRI room to minimize head motion.

With respect to the task‐based fMRI, the task component consisted of two fMRI runs in which participants were presented with 20 persuasive messages (10 affective and 10 cognitive) about various consumer products. The messages were randomized and presented in a self‐referential format to enhance the differentiation between affective and cognitive content, as validated in preliminary studies.

Task runs incorporated two phases. In the first phase, the reading phase, participants were shown each persuasive message and were instructed to read each message. The affective and cognitive messages were presented in a randomized order in the two fMRI runs. The duration for the reading phase was set on the basis of the pretest that was conducted to ascertain that the time for the reading was sufficient for participants and to ascertain that the affective and cognitive messages did not differ in duration. This reading time was also adapted to be a multiple of the TR (i.e., 1800 ms; the average reading time across the messages was 34,960 ms). In the second phase, the evaluation phase, which followed each message after a randomly varying interval (1.8–5.4 s), participants provided their evaluations. Such evaluations included two ratings: an Attitude Rating, for which participants rated their liking of the presented object on a scale from 1 (not at all) to 7 (very much), and an Intention Rating, where participants indicated their likelihood of purchasing the object within the next 3 weeks on a scale from 1 (not at all) to 7 (very likely).

Responses were made using a response pad, with participants adjusting their ratings by pressing buttons to increase or decrease the score from a starting value of 4. Each evaluation had a time limit of 5.4 s. Given the high correlation between attitude and intention ratings (*r* = 0.96, *p* < 0.001), these responses were averaged to create a single index labeled Evaluation, representing the extrinsic behavioral measure in this study.

### 2.6 MRI Data Preprocessing

Functional images were preprocessed and analyzed using AFNI software (Cox 1996). The preprocessing pipeline included deobliquing, despiking, and correcting for time‐shifted acquisition. Motion correction was performed using a six‐parameter model and body realignment, followed by nonlinear warping to align the images to the Montreal Neurological Institute (MNI) standard brain template. Motion parameters were recorded to account for movement in subsequent analyses. BOLD signals were scaled to a mean of 100 to represent percent signal change, enhancing interpretability (Chen et al. 2017). Finally, images were spatially smoothed with a 5‐mm full width at half maximum (FWHM) Gaussian filter. All processing steps, including motion correction, nuisance regression (motion parameters, WM, CSF, and drift terms), and stimulus timing verification, followed standardized guidelines. To ensure rigorous quality control, we adhered to the multi‐stage hierarchical QC procedures recommended by Reynolds et al. (2023), including quantitative motion metrics, visual inspection, and outlier detection using AFNI's afni_proc.py pipelines. No participants were excluded due to excessive motion (i.e., > 20% censored volumes).

#### 2.6.1 Processing MRI Data: Intrinsic Data (Resting‐State)

With respect to the resting‐state runs, and in line with current guidelines (Power et al. 2013), time series were additionally censored by removing volumes with 10% or more motion outliers across voxels and volumes with Euclidean norm of the motion derivative exceeding 0.2 mm. A band‐pass filter (frequency interval: 0.01–0.10 Hz) was applied in the same regression step that implemented censoring (Caballero‐Gaudes and Reynolds 2017). To maximize signal‐to‐noise ratio, motion parameters were included in the regression as noise covariates together with the signals extracted from white matter and cerebrospinal fluid. We did not regress out the global signal because it is a controversial approach (Saad et al. 2012), and because it has been shown that it introduces spurious negative correlations (Wasserstein et al. 2019).

#### 2.6.2 Processing MRI Data: Extrinsic Data (Task‐Evoked Activity)

Task runs were additionally analyzed by implementing a generalized linear model (GLM) at the single‐subject level to estimate brain‐evoked activity during the affective and cognitive conditions of the task. The GLM was implemented in AFNI and included two regressors of interest representing the affective and cognitive experimental conditions, which were modeled with duration‐modulated BLOCK functions. The duration of the BLOCK function for each trial corresponded to the duration calculated for each target during the pilot experiments. Keypresses for target evaluations were modeled through separate regressors using GAM functions. Each GLM also included the following regressors of no interest: six parameters of motion regressors (x‐axis, y‐axis, z‐axis, yaw, pitch, and roll), cerebrospinal fluid signal, white matter signal, linear and non‐linear drifts. Once the brain activity was estimated in each experimental condition, we calculated the difference Δβ_A‐C_ = β_A_—β_C_, where β_A_ is the value for the regressor Affective and β_C_ is the value for the regressor Cognitive. Thus, the term Δβ_A‐C_ represents the difference in evoked activity between affective and cognitive persuasive stimulation and was used in later analysis steps. We also adopted a single‐trial modeling of brain activity (Pessoa 2008; Chen et al. 2021) to allow the extraction of Δβ*i*_A‐C_ related to each target *i* to gather trial‐level information to be implemented in machine learning models (see below). The metric of (differential) task‐evoked activity represents the extrinsic feature of the brain in our study.

### 2.7 Resting‐State Connectomics

Resting‐state fMRI runs were utilized to extract modular structures (brain functional networks) and calculate graph indices from functional connectivity matrices. The brain was parcellated into 386 cortical and subcortical nodes based on Joliot et al. (2015) and 32 cerebellar nodes from the Diedrichsen atlas (2009), resulting in a total of 418 nodes. Functional connectivity between each pair of nodes was assessed using the Pearson correlation of their average time series, using the Fisher z‐score for normalization. Participant‐level functional connectivity matrices were binarized using a proportional threshold, retaining the top 10% of strongest correlations. This approach is widely used in network neuroscience to ensure comparability across participants and to preserve the interpretability of graph metrics (Rubinov and Sporns 2010; van Wijk et al. 2010). The 10% threshold allows for the retention of strong and meaningful connections while minimizing the inclusion of noise‐driven edges (Garrison et al. 2015).

Modular structures were identified using the Louvain algorithm (Lancichinetti and Fortunato 2009) implemented in the Brain Connectivity Toolbox (Rubinov and Sporns 2010) within MATLAB (version 2019b). This process involved 1000 iterations to account for the algorithm's stochastic nature, resulting in a consensus modular structure at the group level using a community detection approach (Lancichinetti and Fortunato 2009). The structural resolution parameter γ, crucial for network analysis (Betzel et al. 2016), was varied systematically between 0.3 and 5.0 to explore its impact on network modularity. Significant modules within the consensus structure were detected using the Newman–Girvan procedure (Newman and Girvan 2004).

For each node, two graph metrics were extracted to characterize network integration and segregation: the participation coefficient (indicating the extent of a node's connections across different modules) and the within‐module degree (reflecting the strength of a node's connections within its own module). These metrics serve as intrinsic features of brain connectivity in this study. To examine the relationships between brain connectivity and behavioral measures, group‐level analyses were conducted for each detected module across all γ values. This comprehensive approach allows for the assessment of how intrinsic brain network properties relate to affective‐cognitive orientations and their association with responses to persuasive messages.

### 2.8 Analysis of Extrinsic Brain‐Behavior Relationships

Task‐evoked neural activity correlates of subjective persuasive message evaluations were assessed using mixed‐effects regression models. We implemented a regression model to assess if the difference in evoked activity between affective and cognitive persuasive stimulation (Δβ_A‐C_) corresponded with differentially favorable affective judgments (i.e., when a product is associated with an affective appeal) relative to cognitive judgments (i.e., when a product is associated with a cognitive appeal)—our Evaluation measure. To note, the individual Evaluation, which incorporated attitude and intention, considers how individuals evaluated the same product in response to affective versus cognitive presentation (i.e., if they preferred the object when it was introduced by an affective or a cognitive presentation). The analysis was carried out at the whole‐brain level and using the parcellations described in the resting‐state section (Δβ_A‐C_ values were averaged within voxels of the same node). Both random intercepts and slopes were added at the nodal level in the mixed‐effects regression model to investigate which brain regions/hemisphere reflect individuals' judgments. We reported standardized coefficients alongside raw estimates to facilitate interpretation and allow interpretability across studies, acknowledging that model structure and data hierarchy have a great impact on data analysis (see Brysbaert and Stevens 2018; Westfall et al. 2014).

### 2.9 Analysis of Rest‐Task Interplay

Once we assessed the resting‐state neural correlates of Orientation and task‐evoked neural correlates of Evaluation, we studied the interplay between these two sets of state‐dependent features. More precisely, we assessed if intrinsic (network architecture and Orientation) brain‐behavior background may predict extrinsic (task‐evoked activity and Evaluation) brain‐behavior features. To achieve this aim, we implemented both a regression approach and a cross‐validated machine learning model.

To study such interactions through regression, we broke down the task‐rest interplays into three models: a “*brain*” model, a “*behavior*” model, and a “*brain‐behavior*” model. In the models, task measures were considered as dependent variables. We found this approach to be more interpretable and parsimonious than other methods (e.g., partial least squares). Thus, the *brain model* assessed the associations between the brain variable of interest during the extrinsic task (Δβ_A‐C_) and the brain variable of interest during the intrinsic resting state (participation coefficients, within‐module degrees). The *behavior model* assessed the associations between the extrinsic behavioral variable of interest (Evaluation) and the intrinsic behavioral variable of interest (Orientation). Finally, the *brain‐behavior model* assessed if the brain‐behavior interplay (i.e., the linear product between nodal Δβ_A‐C_ and subjective Evaluation) was predicted by resting‐state brain metrics, by Orientation, and/or by the interaction between these two terms. To note, since the dependent variable is the product of subjective Evaluation and Δβ_A‐C_, it has positive (> 0) values when there is congruence between evoked activity and Evaluation, whereas it has negative (< 0) values when there is incongruence between evoked activity and Evaluation. Combining the three models is appropriate and necessary, as it provides more information than studying, for example, brain‐behavior interactions alone.

We adopted a combined approach based on both traditional regression models and cross‐validated machine learning to ensure a robust and interpretable characterization of rest‐task relationships. This hybrid strategy allowed us to complement the explanatory power and transparency of regression modeling with the generalization potential of machine learning, enhancing both the inferential and predictive value of our analyses.

It is important to note that our analytical framework leverages hierarchical mixed‐effects models, which offer increased statistical power relative to standard linear models by appropriately accounting for within‐subject and within‐region variability. This approach is well‐supported in the literature as particularly suitable for neuroimaging designs involving repeated measures and high‐dimensional data (Chen et al. 2017; Westfall et al. 2014; Mumford and Nichols 2008). To empirically validate the sensitivity of our pipeline, we conducted a simulation‐based power analysis tailored to the structure of our models. We generated synthetic datasets that matched our design (i.e., number of subjects, number of ROIs, and model hierarchy) and tested the ability of our mixed‐effects models to detect fixed effects of varying magnitudes. Results indicated that, with our current sample size and modeling structure, the mixed‐effects framework was able to detect small effects (β = 0.020) with approximately 75% power, medium‐small effects (β = 0.050) with over 95%power, and medium‐large effects (β = 0.010) with 100% power. These findings support the adequacy of our design for capturing even subtle brain‐behavior relationships.

## 3 Results

### 3.1 Extrinsic Brain‐Behavior Relationships

**FIGURE 1:**
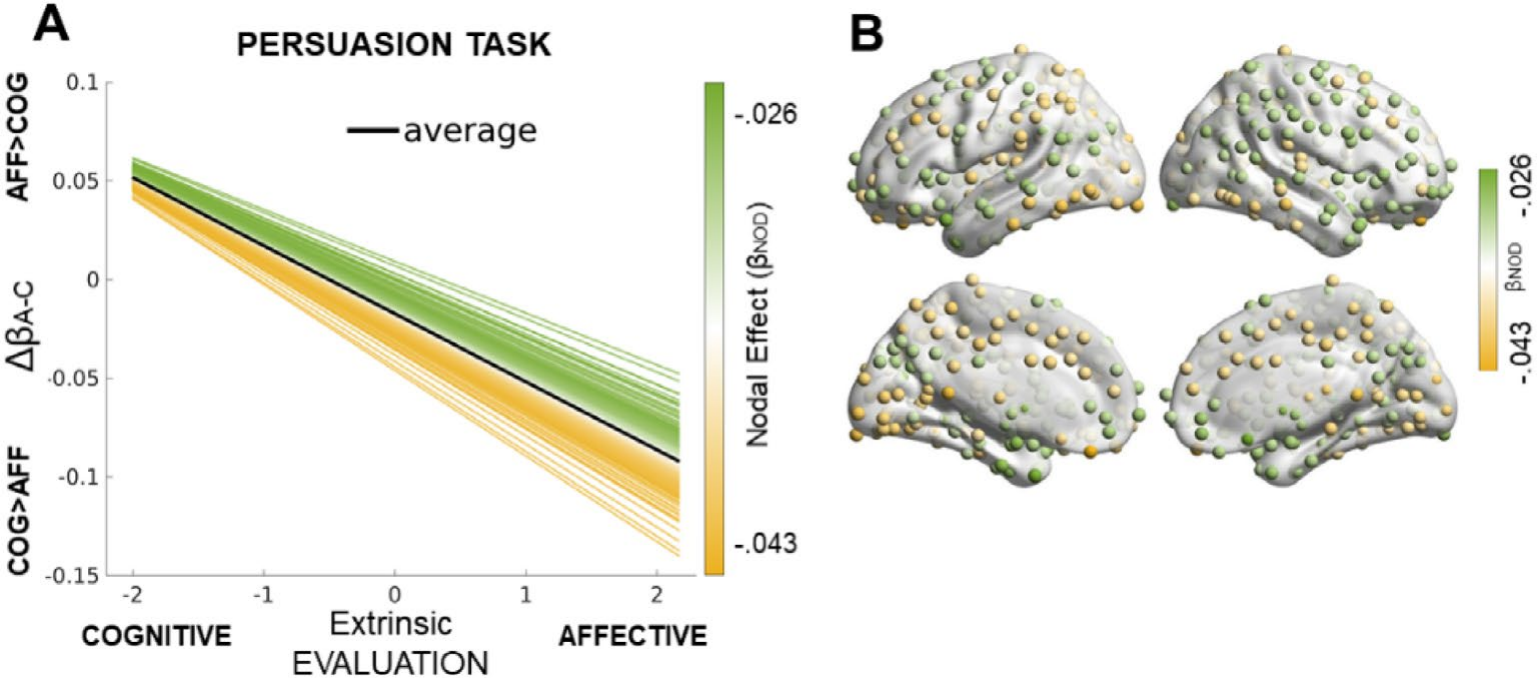
Task results. (A) The difference in evoked activity across conditions (Δβ_A‐C_) at the whole‐brain level was negatively associated with extrinsic evaluation. Each brain node is represented by a separate line and color‐coded based on nodal effect sizes (β_NOD_). (B) Topography and nodal effect sizes (β_NOD_) related to the association between task‐evoked activity and Evaluation. The color coding is the same across the two subfigures, showing a slightly weaker effect in the right hemisphere and a slightly stronger effect in the left hemisphere. The results show that brain activity was generally higher when the Evaluation was incongruent with the experimental condition (e.g., greater Evaluation for the affective target, during the Cognitive condition).

We first examined the relationship between neural responses during persuasion (i.e., task‐evoked activity) and participants' behavioral evaluations of targets introduced by affective versus cognitive messages. Specifically, we focused on the differential neural activation between affective and cognitive persuasive conditions (Δβ_A–C_), interpreted as an index of evaluative congruence or incongruence. A significant negative association was observed between the difference in task‐evoked activity across affective and cognitive experimental conditions (Δβ_A–C_) and subjective evaluation (Figure 1A; β = −0.034 ± 0.012, standardized β = −0.167, *t* = −2.78, *p* = 0.006).

This result indicates that greater differences in brain activity between the affective and cognitive conditions were associated with lower evaluations, suggesting that incongruence between the type of target and its evaluation is linked to heightened neural responses. In other words, when neural responses strongly diverge between affective and cognitive messaging, participants are more likely to exhibit lower persuasive impact.

Importantly, this effect was consistent across regions, with no significant variability among ROI slopes (Figure 1B), suggesting a global evaluative pattern across the brain. However, a hemispheric effect was identified: the association tended to be slightly stronger in the left hemisphere (yellow nodes in Figure 1B), whereas a slightly weaker effect was detected in the right hemisphere (green nodes in Figure 1B). These hemispheric differences, while modest, suggest lateralized processing differences during task‐related evaluations.

### 3.2 Intrinsic‐Extrinsic Interplay

To examine how intrinsic brain network properties modulate persuasive processing, we tested three interconnected models capturing the brain, behavior, and brain‐behavior relationships across rest and task.

The first model (brain model) tested whether intrinsic connectivity, as captured by the participation coefficient (PC), predicts task‐evoked neural responses (Δβ_A‐C_). In the brain model, we did not observe a significant association between the difference in task‐evoked activity and participation coefficients (β = 0.002 ± 0.004, *t* = 0.53, *p* = 0.59), indicating that intrinsic network participation alone does not predict task‐evoked brain activity differences.

Similarly, in the behavioral model, Orientation (a continuous index of cognitive‐affective motivational orientation) did not directly predict Evaluation (β = 0.024 ± 0.091, *t* = 0.27, *p* = 0.79), suggesting no main behavioral preference for affective or cognitive messages across the sample.

However, in the brain‐behavior model, we identified significant associations. Specifically, both nodal participation coefficient (β = −0.008 ± 0.002, *t* = −3.68, *p* < 0.001) and the interaction between Orientation and nodal participation coefficient (β = 0.010 ± 0.002, *t* = 4.33, *p* < 0.001) were significant, while Orientation alone was not (β = 0.009 ± 0.008, *t* = 1.25, *p* = 0.21). In other words, only when individuals had high cross‐network integration in a given region (high PC) did their motivational orientation (affective vs. cognitive) significantly modulate the alignment between neural responses and evaluations. These results indicate that the strength of cross‐network participation (as measured by the participation coefficient) and its interaction with individual orientation are critical for understanding the relationship between resting‐state brain properties and task‐related evaluations. The results from the three models, as well as standardized coefficients, are represented in Table 1.

**TABLE 1:** Results for the system of regressions implemented to examine the relationship between intrinsic and extrinsic brain‐behavior features.

Term	Effect size (β)	Standard error	$\hat{\beta }$	*t*‐Stat	*p*
*1—Brain Model:* Δβ ~ PC + (1|node) + (1|subject)					
Intercept	−0.018	0.008	—	−2.27	0.02
PC	0.002	0.004	0.005	0.53	0.59
*2—Behavior Model:* Evaluation ~ Orientation					
Intercept	0.007	0.097	—	0.07	0.94
Orientation	0.024	0.091	0.011	0.27	0.79
*3—Brain‐Behavior Model*: Δβ:Evaluation ~ PC*Orientation + (1|node) + (1|subject)					
Intercept	−0.008	0.008	—	−0.98	0.02
**PC**	−0.008*	0.**002**	**−0.024**	**−3.68**	**< 0.001**
Orientation	0.009	0.008	0.133	1.25	0.21
**PC:Orientation**	0.**010**	0.**002**	0.**058**	**4.33**	**< 0.001**

*Note:* Brain Model. This model assesses the association between brain connectivity (Δβ) and participation coefficient (PC), a measure of intrinsic connectivity, controlling for random effects by node and subject. Results indicate a significant intercept (β = −0.018, *p* = 0.02), suggesting baseline brain activity levels, but no significant effect of PC on Δβ (*p* = 0.59), implying that the participation coefficient alone does not predict brain connectivity changes in this context. Behavior Model. This model explores how individual orientation (NFA or NFC) predicts evaluation responses to persuasive messages. Neither the intercept (*p* = 0.94) nor orientation (*p* = 0.79) reaches significance, indicating that orientation alone does not significantly influence evaluation responses. Brain‐Behavior Model. This integrated model examines the interaction between brain connectivity (PC) and orientation in predicting evaluation responses, controlling for random effects by node and subject. Here, PC shows a significant negative effect (β = −0.008, *p* < 0.001), and the interaction between PC and Orientation is also significant (β = 0.010, *p* < 0.001), indicating that PC modulates evaluation responses based on individual orientation. This suggests that brain‐behavior alignment, particularly the interplay between intrinsic connectivity and orientation, significantly influences evaluation, supporting the hypothesis of affective‐cognitive matching at the neural level.

Overall, these models illustrate that while individual components (PC or Orientation) alone may not strongly predict evaluation responses, their interaction in the brain‐behavior model significantly impacts evaluation, emphasizing the role of brain‐behavior alignment in persuasion processes.

**FIGURE 2:**
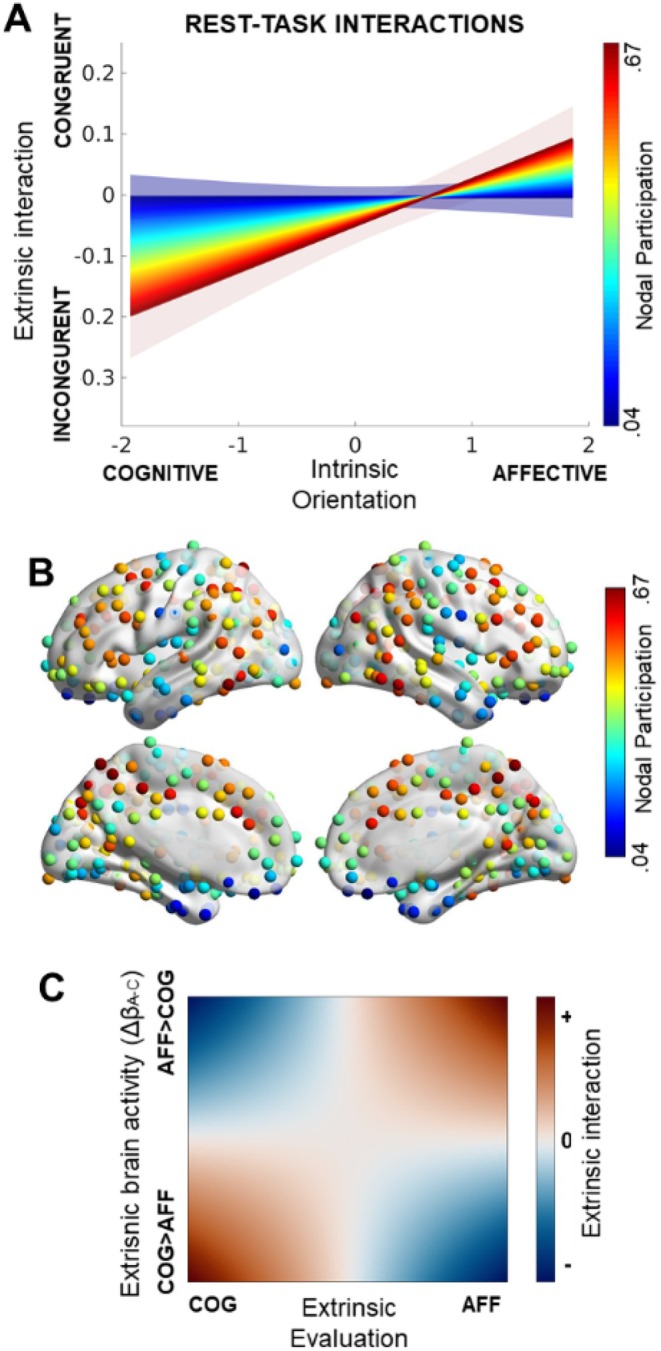
Rest‐task interplay. (A) Predictions of the extrinsic‐interaction term (dependent variable) from the brain‐behavior model linking intrinsic orientation, intrinsic nodal participation, extrinsic Evaluation, and extrinsic brain activity. The system of regression models adopted to study task‐rest associations showed a significant effect of the interaction orientation *participation coefficient on extrinsic brain‐behavior data. (B) Nodes with high participation coefficients in our study (red) largely overlap with the FP network (see Di Plinio et al. 2023; Power et al. 2013), encompassing prefrontal, inferior parietal, mid‐cingulate, and posterior temporal nodes. Instead, nodes with low participation (low) include limbic and sensory regions. (C) The dependent variable of interest, namely the extrinsic brain‐behavior interaction, represents the linear product between evaluation and the difference in task activity across conditions (Δβ_A‐C_). These results indicate that cognitive‐oriented individuals are likely to have greater brain activity in nodes with high participation coefficients (e.g., fronto‐parietal network) when the experimental condition is incongruent with their evaluation. Instead, affective‐oriented individuals are likely to have greater brain activity in nodes with high participation coefficients when the experimental condition is congruent with their evaluation.

To better understand the significance in the brain‐behavioral model, Figure 2A depicts the model predictions of the interaction effect between intrinsic (rest) and extrinsic (task) brain‐behavior data: in nodes with low participation coefficients (intense blue), there is no significant effect of Orientation on extrinsic brain‐behavior interactions. Conversely, in nodes with high participation coefficients (intense red), a positive effect of Orientation on extrinsic brain‐behavior interactions is observed.

The participation coefficient map (Figure 2B) aligns with previously reported findings (Power et al. 2013), showing high values in prefrontal, cingulate, inferior parietal, and posterior temporal nodes. These regions largely overlap with the fronto‐parietal (FP) subnetwork, which has been previously associated with Orientation during the resting state (Di Plinio et al. 2023). Additionally, since the dependent variable represents the linear product between Evaluation and the difference in task‐evoked activity across conditions (Δβ_A‐C_), higher values (> 0) indicate matching (*congruence*) between evoked activity and Evaluation, whereas lower values (< 0) reflect incongruence between evoked activity and Evaluation (Figure 2C).

Summarizing, among affective‐oriented individuals (standardized Orientation > 0), FP nodes with high participation coefficients are more activated when the type of stimulus aligns with the individual's evaluation (i.e., when the stimulus has affective features). In contrast, among cognitive‐oriented individuals (standardized Orientation < 0), the same high‐participation nodes are involved when there is incongruence between the experimental condition and the individual's evaluation (e.g., an affective condition paired with a higher cognitive evaluation, or vice versa). Notably, these effects only apply to regions with high cross‐network connections (e.g., FP regions), but not to regions with low cross‐network connections (e.g., sensory or limbic areas). No significant results were found for within‐module degrees.

## 4 Discussion

This study explored how intrinsic brain connectivity interacts with extrinsic task‐related neural and behavioral responses within the affective‐cognitive framework of persuasion.

We report two particularly novel and important results. First, we found a significant negative association between task‐evoked brain activity (Δβ_A‐C_) and subjective evaluations, indicating that when the message is incongruent with its evaluation, neural responses are more intense, particularly in the left hemisphere. Second, whereas intrinsic cross‐network connectivity (participation) and affective‐cognitive orientation did not independently predict extrinsic outcomes, their interaction was significantly associated with those outcomes. High participation coefficients within regions of the frontoparietal network were linked to evaluations that varied as a function of individuals' affective‐cognitive orientation. In affective‐oriented individuals, high participation nodes were more active during congruent conditions, whereas in cognitive‐oriented individuals, the same nodes were more active during incongruent conditions.

These findings extend the affective‐cognitive matching effect (Edwards 1990; Fabrigar and Petty 1999; Haddock et al. 2008) to a neural level, showing that intrinsic brain connectivity modulates responses to persuasive messages. This integration provides a deeper understanding of the mechanisms underlying personalized persuasion, highlighting the role of frontoparietal hubs in aligning brain connectivity with motivational orientations.

### 4.1 Extrinsic Brain‐Behavior Relationships

The negative association between extrinsic brain activity (Δβ_A‐C_) and subjective evaluations supports the affective‐cognitive matching effect (Petty and Cacioppo 1986; Fabrigar and Petty 1999; Haddock et al. 2008), where cogent persuasive messages have a greater impact when aligned with the recipient's affective or cognitive orientation. Specifically, our findings show that mismatched message types increase neural activity; in other words, incongruent conditions may trigger additional neural resources, potentially causing cognitive strain. This aligns with evidence demonstrating that the brain recruits broader networks to handle increased cognitive demands (Botvinick et al. 2004; Shenhav et al. 2013). The fronto‐parietal network, involved among others in functions as cognitive control and maintaining task goals (Cole and Schneider 2007; Dosenbach et al. 2008), may play a role in reconciling message‐evaluation mismatches. Thus, the increased neural activation we observe in response to mismatched persuasive messages is consistent with broader models of neural economy, where greater resource expenditure is required to maintain performance under conditions of cognitive conflict (Poldrack et al. 2009).

The negative association predominantly observed in the left hemisphere reflects its putative role in analytical and cognitive processing, explaining why incongruent cognitive messages tend to elicit greater activation in this region (Corser and Jasper 2014). This lateralization is supported by studies demonstrating the left hemisphere's critical involvement in managing complex cognitive tasks and resolving cognitive‐emotional conflicts (Gainotti 2019). Our findings underscore the importance of aligning persuasive message content with individual affective or cognitive orientations. Increased neural resource allocation during mismatched conditions underscores the cognitive effort needed to process incongruent information. Our findings align with earlier work demonstrating continuity between resting and task‐related functional architectures (Smith et al. 2009), while also supporting the view that dynamic reconfigurations—particularly within integrative hubs such as the frontoparietal network—are sensitive to context‐specific cognitive engagement (Utevsky et al. 2014). This dual nature underscores the potential of rest‐task integration in capturing both trait‐like and state‐dependent components of persuasion processing.

### 4.2 Intrinsic‐Extrinsic Interplay

The interaction between participation coefficients and Orientation supports the affective‐cognitive matching effect by facilitating cross‐network integration. Previous studies have shown that high participation nodes aid cognitive control and attentional modulation by enabling efficient communication across networks (Dosenbach et al. 2008; Menon 2011). As applied to the present research, in affective‐oriented individuals, increased activity in high participation nodes during congruent conditions suggests effective processing of matched persuasive messages. Conversely, cognitive‐oriented individuals show this increased activity during incongruent conditions, indicating efforts to resolve cognitive discrepancies. Such increased neural recruitment suggests enhanced analytical processing to resolve cognitive discrepancies (Bunge et al. 2009). The regions involved (e.g., supramarginal gyrus, dorsolateral prefrontal cortex) are implicated in higher‐order cognitive functions, including error detection and conflict resolution, which are essential for resolving incongruent information and adapting behavioral responses (Nee and D'Esposito 2016).

These findings highlight the crucial role of high participation nodes, or hubs, in facilitating network integration during complex evaluations by integrating affective and cognitive inputs (Barrett and Satpute 2013; Pessoa 2008). This type of top‐down regulation may enable affective‐oriented individuals to align their intrinsic predispositions with external affective cues, thereby enhancing the persuasive impact of congruent messages (Ochsner and Gross 2005). This perspective supports the notion that intrinsic brain networks may be functionally aligned with the brain's response to external tasks, consistent with frameworks proposing that intrinsic connectivity patterns scaffold task‐related neural responses (Di Plinio et al. 2023; Tavor et al. 2016). These findings are also consistent with research suggesting that intrinsic network properties are associated with individual differences in how people engage with task‐related demands, including conflict processing (Fox and Raichle 2007; Raichle 2015).

Our study provides a comprehensive view of how intrinsic brain connectivity relates to motivational orientations and how this relationship is associated with responses to persuasive messages. By integrating graph‐theoretical metrics that quantify cross‐network integration, we offer a deeper look into the neural architecture underlying individual differences in evaluative processing. This advancement contributes to bridging psychological models with large‐scale brain network dynamics. By demonstrating that the interaction between intrinsic network properties and individual orientation is associated with variability in extrinsic evaluations, our findings refine our understanding of the affective‐cognitive matching effect.

### 4.3 Implications for Persuasive Communication

Understanding the neural basis of the affective‐cognitive matching effect offers significant insights for designing persuasive messages. By aligning message content with the target audience's intrinsic affective or cognitive orientations, communicators can enhance message self‐relevance and processing efficiency. For example, a health promotion initiative might use affectively oriented messages that emphasize the emotional well‐being associated with physical activity for affective‐oriented individuals, while presenting statistical evidence about the health benefits of exercise for cognitive‐oriented individuals, thereby increasing persuasive impact and consumer engagement (see Maio et al. 2018). Our findings extend the affective‐cognitive matching effect by showing that intrinsic brain connectivity interacts with individual orientations and is associated with differences in neural and behavioral responses to affective and cognitive information, suggesting that intrinsic brain networks may contribute to the alignment between motivational predispositions and external persuasive stimuli. This integration bridges psychological and neuroscientific perspectives, offering a more comprehensive view of personalized persuasion.

These results resonate with the ELM (Petty and Cacioppo 1986; Petty et al. 2015), which posits that persuasion occurs through central and peripheral routes based on an individual's motivation and ability to process information. The intrinsic‐extrinsic interplay suggests that self‐relevance, driven by message‐content matching, increases the likelihood of central processing, possibly leading to more enduring attitude change compared to peripheral processing. Furthermore, the role of frontoparietal hubs in mediating this interplay underscores the importance of neural resource allocation in persuasive communication. High participation coefficients within these regions facilitate cross‐network integration, enabling efficient processing of congruent persuasive messages in affective‐oriented individuals and the reconciliation of incongruent messages in cognitive‐oriented individuals.

Additionally, our study highlights the novelty of integrating intrinsic brain connectivity with extrinsic task‐related responses to understand processes underlying persuasion. This approach advances psychological models by incorporating neural predispositions, offering a more nuanced framework for predicting and enhancing persuasive outcomes. Future psychological models of persuasion should consider both intrinsic neural architectures and extrinsic processing dynamics to better capture the complexity of attitude formation and change. While our findings were derived from a consumer context, the implications of affective‐cognitive matching extend well beyond marketing applications. Indeed, tailoring persuasive content to match individuals' intrinsic motivational orientations could be equally valuable in domains such as health promotion, environmental communication, and political campaigns. For example, individuals with high NFA might respond more favorably to emotionally charged public health messages (e.g., emphasizing hope, fear, or empathy; Heffner et al. 2021) whereas those with high NFC may be more persuaded by messages grounded in data, logic, and critical reasoning (Aquino et al. 2020; Conner et al. 2021).

In addition, affective‐cognitive matching can be used to address the challenges of contemporary society. For example, discussions surrounding global warming have historically been dominated by the debate about the existence of the effect and the role of human beings in generating it. Research on the affect‐cognition matching effect shows that engagement with the underlying emotion of an attitude can be at least as important as engagement with cognition (see also Rocklage and Luttrell 2021). With the increase of interventions involving citizens, institutions, and companies in the process of change, research that identifies the role of these matching effects can help to build messages that are more likely to be personally relevant and accessible to the target audience, allowing for better dialogue on global issues.

Future research should empirically test the generalizability of the affective‐cognitive matching effect across such domains, using content‐specific persuasive stimuli and outcomes that reflect real‐world behaviors. This would help establish whether the neural and psychological mechanisms identified here operate similarly across contexts, or whether they interact with domain‐specific variables such as topic relevance, message credibility, or social norms.

Overall, these findings not only reinforce existing theories of persuasion but also provide actionable strategies for enhancing persuasive communication. By leveraging neuroscientific insights, practitioners can develop more targeted and effective persuasive messages that resonate deeply with individual orientations, ultimately fostering meaningful and lasting attitude change across various domains such as health promotion, sustainability, marketing, and education.

### 4.4 Limitations and Future Directions

While our study provides valuable insights, several limitations must be acknowledged. First, our sample consisted of healthy Italian adults, which may limit the generalizability of the findings. Second, the reliance on self‐reported measures of evaluation could potentially introduce biases such as social desirability or response consistency. Future studies should supplement self‐report measures with objective behavioral metrics to enhance the validity of behavioral assessments. Third, the stimuli were limited to positively valenced consumer messages, restricting the applicability of the findings to other contexts and emotional tones. Future research can explore a broader range of message types and domains to assess the robustness of the affective‐cognitive matching effect. Fourth, the present study focused primarily on spatial patterns of brain activity and static connectivity measures, without directly addressing the temporal dynamics of persuasive processing. However, emerging approaches in dynamic functional connectivity offer promising tools to capture fluctuations in network organization over time, which may align with transitions across stages of message reception, evaluation, and internalization. Integrating spatial and temporal neural features would offer a more comprehensive understanding of the mechanisms by which intrinsic brain states influence evaluative processing in real time.

Finally, our design is inherently correlational, which limits causal inference. While our results suggest meaningful associations between intrinsic brain states, motivational orientations, and persuasive outcomes, future studies should consider experimental manipulations to establish causal links. For example, non‐invasive neuromodulation techniques such as transcranial magnetic stimulation (TMS) or transcranial direct current stimulation (tDCS) could be used to transiently modulate network‐level properties (e.g., frontoparietal connectivity) and assess their effect on evaluative responses. Additionally, interventions aimed at altering motivational orientations (e.g., through priming or framing tasks) could be employed to observe changes in persuasion outcomes. These approaches would help clarify the directionality of the observed relationships and strengthen the mechanistic interpretation of the affective‐cognitive matching effect.

### 4.5 Conclusion

This study advances our understanding of the neural mechanisms underlying the affective‐cognitive matching effect by demonstrating that intrinsic brain connectivity patterns of individuals interact with their psychological affective‐cognitive orientations to influence task‐related evaluations. Functional brain regions with high cross‐network communication in the fronto‐parietal network emerge as critical hubs for aligning intrinsic predispositions with extrinsic evaluative processes, highlighting the importance of cross‐network integration in persuasive communication. Importantly, while our study focused on consumer‐related persuasive messages, the underlying mechanisms are likely relevant to a broad array of contexts—including health, environmental, and political communication—where attitude change is a key outcome. Future work should explore these domains to test the cross‐contextual robustness and translational potential of affective‐cognitive matching in both research and applied communication strategies.

## Author Contributions

**S. Di Plinio:** conceptualization, data curation, formal analysis, methodology, software, visualization, writing – original draft, writing – review and editing. **A. Aquino:** conceptualization, data curation, writing – original draft, writing – review and editing. **G. Haddock:** writing – review and editing. **F. R. Alparone:** project administration. **S. J. H. Ebisch:** conceptualization, methodology, project administration, supervision, writing – review and editing.

## Conflicts of Interest

The authors declare no conflicts of interest.

## Data Availability

Anonymized summary statistics and analysis scripts used for preprocessing, modeling, and visualization are available upon reasonable request to the corresponding author.
